# Anti-SARS-CoV Natural Products With the Potential to Inhibit SARS-CoV-2 (COVID-19)

**DOI:** 10.3389/fphar.2020.561334

**Published:** 2020-09-25

**Authors:** Surjeet Verma, Danielle Twilley, Tenille Esmear, Carel B. Oosthuizen, Anna-Mari Reid, Marizé Nel, Namrita Lall

**Affiliations:** ^1^ Department of Plant and Soil Sciences, Faculty of Natural and Agricultural Sciences, University of Pretoria, Pretoria, South Africa; ^2^ School of Natural Resources, College of Agriculture, Food and Natural Resources, University of Missouri, Columbia, MO, United States; ^3^ College of Pharmacy, JSS Academy of Higher Education and Research, Mysuru, India; ^4^ Bio-Tech R&D Institute, University of the West Indies, Kingston, Jamaica

**Keywords:** ****coronavirus, COVID-19, ethnomedicine, HCoV, natural products, novel drug candidates, SARS-CoV, viral infections

## Abstract

The severe acute respiratory syndrome coronavirus-2 (SARS-CoV-2), known to cause the disease COVID-19, was declared a pandemic in early 2020. The objective of this review was to collate information regarding the potential of plants and natural products to inhibit coronavirus and targets associated with infection in humans and to highlight known drugs, which may have potential activity against SARS-CoV-2. Due to the similarity in the RNA genome, main proteases, and primary host receptor between SARS-CoV and SARS-CoV-2, a review was conducted on plants and secondary metabolites, which have shown activity against SARS-CoV. Numerous scientific reports on the potential of plants and secondary metabolites against SARS-CoV infection were found, providing important information on their possible activity against SARS-CoV-2. Based on current literature, 83 compounds have been identified with the potential to inhibit COVID-19. The most prominent selectivity was found for the alkaloid, lycorine, the lignan, savinin, and the abietane terpenoid, 8-beta-hydroxyabieta-9(11),13-dien-12-one with selectivity index values greater than 945, 667, and 510, respectively. Plants and their secondary metabolites, with activity against targets associated with the SARS-CoV infection, could provide valuable leads for the development into drugs for the novel SARS-CoV-2. The prospects of using computational methods to screen secondary metabolites against SARS-CoV targets are briefly discussed, and the drawbacks have been highlighted. Finally, we discuss plants traditionally used in Southern Africa for symptoms associated with respiratory viral infections and influenza, such as coughs, fever, and colds. However, only a few of these plants have been screened against SARS-CoV. Natural products hold a prominent role in discovering novel therapeutics to mitigate the current COVID-19 pandemic; however, further investigations regarding *in vitro*, *in vivo*, pre-clinical, and clinical phases are still required.

## Introduction

Severe acute respiratory syndrome coronavirus (SARS-CoV) is a highly contagious viral infection that causes considerable morbidity and mortality (Simmons et al., 2004). The SARS-CoV is part of the family Coronaviridae, which are enveloped viruses with single and positively stranded RNA (Du et al., 2009). This virus is known to cause respiratory, enteric, and neurological diseases in humans (Simmons et al., 2004). It is one of seven coronaviruses that have been shown to cause human infection. This includes the novel SARS-CoV-2, which is responsible for causing the coronavirus disease of 2019 (COVID-19). Other coronaviruses include the alpha coronaviruses (HCoVs-NL-63 and HCoVs-229E) and the beta coronaviruses [HCoVs-OC43, HCoVs-HKu1, Middle East respiratory syndrome-CoV (MERS-CoV), and SARS-CoV]. The COVID-19 outbreak originated from the Wuhan province in China during December 2019. It has developed into a global pandemic in a matter of months, spreading to 214 countries, areas, or territories.

**Figure 1:**
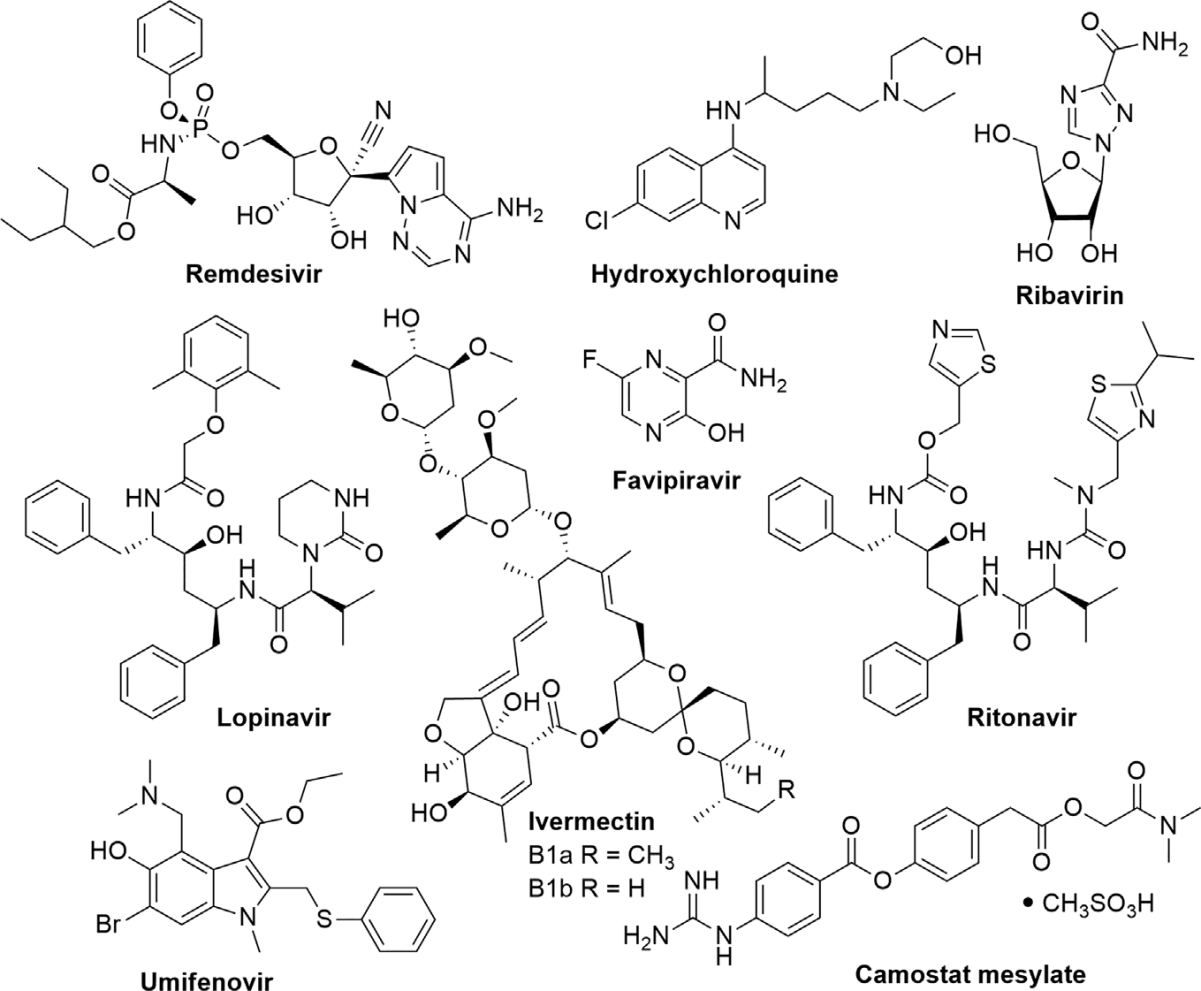
Existing drugs which are being repurposed for the experimental treatment of COVID-19.

Currently, there is no antiviral treatment for COVID-19; therefore, the control of this disease has become a global health emergency. Given the rapid transmission of the virus, researchers and public health agencies are investigating the possibility of repurposing existing drugs for the potential treatment of COVID-19 (**Figure 1**). The WHO is focusing on four promising therapies; an experimental antiviral drug remdesivir (used for the treatment of Ebola), the antimalarial drugs chloroquine and hydroxychloroquine, a combination of two HIV drugs (lopinavir and ritonavir), and the latter combined with interferon-β, an antiviral cytokine and modulator of the immune system (Kupferschmidt and Cohen, 2020).

In a study by Wang et al. (2020), patients admitted to hospital with severe COVID-19, displayed a faster improvement when using remdesivir to those receiving the placebo; however, this was not statistically significant, and it was concluded that larger-scale studies were required to adequately assess the potential therapeutic efficacy of remdesivir. Additionally, remdesivir did not significantly improve mortality or clearance time of the virus (Wang et al., 2020). In a study by Beigel et al. (2020), which was a larger-scale study than conducted by Wang et al. (2020), remdesivir shortened the recovery time in patients that were hospitalized with COVID-19 and showed signs of lower respiratory tract infection compared to recovery times of patients receiving the placebo, from an average of 15 to 11 days; however, it was found that there was no significant difference in mortality rate compared to the placebo control, and therefore, this study concluded that treatment with an antiviral drug alone might not be sufficient in treating COVID-19 (Beigel et al., 2020).

The US food and drug administration (FDA) recently approved the antimalarial drug, hydroxychloroquine, for the experimental treatment of COVID-19 (Cortegiani et al., 2020). Hydroxychloroquine has also been recommended by the Indian Council of Medical Research (ICMR) for the treatment of COVID-19 (Indian Council of Medical Research, 2020). A study reported that the treatment of COVID-19 patients with hydroxychloroquine significantly reduced the viral load, while combining the treatment with azithromycin enhanced the reduction of the viral load when compared to the controls (Gautret et al., 2020). However, due to the urgency of finding a cure for COVID-19, Gautret et al. (2020) published these results using only a small sample size; therefore, results should be confirmed in a larger study (Gautret et al., 2020). Additionally, *in vitro* results regarding the potential of hydroxychloroquine and chloroquine to inhibit SARS-CoV-2 showed that hydroxychloroquine was a more potent inhibitor of SARS-CoV-2 than chloroquine, with 50% effective concentrations (EC_50_) of 0.72 and 5.47 µM, respectively (Yao et al., 2020). However, on the 5^th^ of June 2020, a statement was released by the Chief Investigators of the Randomized Evaluation of COVID-19 therapy (RECOVERY) trial on the use of hydroxychloroquine for COVID-19. The Independent Data Monitoring Committee reviewed clinical trial data that used hydroxychloroquine and concluded that there was no beneficial effect when COVID-19 hospitalized patients were treated with hydroxychloroquine compared to patients which received standard COVID-19 care and, therefore, RECOVERY has stopped enrolling participants for the hydroxychloroquine trials (Horby and Landray, 2020).

A study by Cao et al. (2020), found that patients hospitalized with severe COVID-19 that were treated with lopinavir-ritonavir showed no significant difference compared to patients who received the standard care for COVID-19 (Cao et al., 2020). However, a study by Hung et al. (2020), which investigated the efficacy of a triple combination of interferon beta-1b, lopinavir-ritonavir, and ribavirin in the treatment of hospitalized COVID-19 patients, found that the triple combination treatment was effective in shortening virus shedding and alleviated symptoms in patients with mild to moderate COVID-19 compared to the group treated with lopinavir-ritonavir alone. However, the study lacked the required placebo control and did not include an interferon beta-1b control group in which to compare efficacy (Hung et al., 2020). Therefore, further studies are required to compare the triple combination treatment to that of a placebo group and to establish the role of interferon beta-1b in the efficacy of the triple combination treatment.

A natural product derivative, ivermectin, which is a mixture of two major homologues, ivermectin B1a (>80%) and ivermectin B1b (<20%), is an anti-parasitic natural product that was isolated from a microorganism found in Japanese soil (Crump and Omura, 2011). It is used for the treatment of parasitic infections such as head lice, scabies, river blindness (onchocerciasis), strongyloidiasis, trichuriasis, ascariasis, and lymphatic filariasis (Ottesen and Campbell, 1994). Ivermectin has shown potent *in vitro* activity against SARS-CoV-2. It reduced viral replication by 99.98% within 48 h after treatment with a single dose of 5 µM. The 50% inhibitory concentration (IC_50_) was determined to be ~2 µM (Caly et al., 2020); however, further studies are required to determine the therapeutic potential against COVID-19. **Table 1** and **Figure 1** summarize existing drugs and chemical structures that are being used for the experimental treatment of COVID-19 based on their efficacy in targeting key proteins found on the COVID-19 virus.

**Table 1:** Drug candidates for key proteins during the coronavirus infection process [adapted from Liu et al. (2020)].

Target protein	Drug name	References
Coronavirus main protease 3CL^pro^ (3CL^pro^)	Lopinavir	(Dayer et al., 2017)
Papain-like protease PL^pro^ (PL^pro^)	Lopinavir	(Dayer et al., 2017)
RNA-dependent RNA polymerase (RdRp)	Remdesivir, ribavirin	(Contreras et al., 2002; Gordon et al., 2020)
Viral spike glycoprotein (S protein)	Arbidol (umifenovir)	(Boriskin et al., 2008)
Transmembrane protease, serine 2 (TMPRSS2)	Camostat mesylate	(Hoffmann et al., 2020)
Angiotensin-converting enzyme 2 (ACE2)	Arbidol (umifenovir)	(Boriskin et al., 2008)

This review focuses on natural products, which have shown activity against SARS-CoV, as a selection criterion for potential inhibition of SARS-CoV-2, due to the genome similarity and the similarity in the main protease structure and the primary host receptor between SARS-CoV and SARS-CoV-2 (Chan et al., 2020; Chen and Du, 2020; Zhang et al., 2020b). In addition, the current state of this research topic is briefly discussed, and gaps in the research are identified. Finally, this review discusses the potential use of Southern African medicinal plants, which have traditionally been used for the treatment of symptoms related to respiratory viral infections, and influenza, to inhibit SARS-CoV-2.

## Similarities Between SARS-CoV and SARS-CoV-2

Both the SARS-CoV and the SARS-CoV-2 are considered zoonotic coronaviruses within the genus Betacoronavirus. Coronaviruses are spherical enveloped viruses which range between 100 and 160 nm in diameter. The positive-sense single-stranded RNA genome (27–32 kb), contained in each particle, forms a complex with the nucleocapsid protein (Salata et al., 2019; Kannan et al., 2020). The genome of the novel SARS-CoV-2 was determined to have an 82% nucleotide identity with SARS-CoV. Through phylogenetic analysis, it was found that the membrane, envelope, spike, nucleoprotein, and the orf1a/b polyproteins clustered closely together; however, the orf3b protein encoded a novel short protein (Chan et al., 2020). It was further confirmed that the primary host receptor for SARS-CoV-2 is the human angiotensin-converting enzyme 2 (ACE2), similar as in the case of SARS-CoV (Ou et al., 2020; Rothan and Byrareddy, 2020; Yan et al., 2020). Furthermore, the homology of the spike-receptor binding domain (RBD) sequence between SARS-CoV-2 and SARS-CoV was found to be 76% similar, and the main proteases between the two viruses were closely related (96% identity) (Chen et al., 2020; Lung et al., 2020). Other similarities between SARS-CoV and SARS-CoV-2 include symptom progression and mode of infection. The initial symptoms observed in infected patients are fever, fatigue, and respiratory problems (Wu et al., 2020). Within 8 to 20 days after the initial onset of symptoms, patients suffer from acute respiratory distress syndrome. After 10 days from the onset of symptoms, patients suffer from lung abnormalities (Prompetchara et al., 2020).

During viral infections, the innate immune cells recognise viral RNA through endosomal RNA receptors, cytosolic RNA sensors, and toll-like receptors (TLR) 3 and 7 (Xagorari and Chlichlia, 2008; Ahmadpoor and Rostaing, 2020). Once the virus has been recognized, a cascade occurs, which activates transcription factors such as nuclear transcription factor (NF-κβ). These transcription factors induce the expression of type I interferons (IFN), which binds to an interferon alpha-receptor (IFNAR) (Ivashkiv and Donlin, 2014). This process activates the JAK-STAT pathway, which suppresses viral replication and removes the virus within the body (Fleming, 2016). During SARS-CoV and SARS-CoV-2 viral infection, the RNA enters into a patient’s tissue by binding to the ACE2 receptor, expressed on host cells using the spike glycoprotein (S protein), which contains the receptor-binding domain (RBD) (Hoffmann et al., 2020).

Patients infected with the human coronavirus SARS-CoV-2 undergo what is denoted a “cytokine storm,” where pro-inflammatory cytokines are generated as a result of SARS-CoV-2 infection (Zhang et al., 2020c). Patients who tested positive for the SARS-CoV-2 coronavirus showed an increased level of interleukin-2 (IL-2), IL-7, IL-10, IL-1β, IL-1 receptor agonist (IL-1RA), IL-8, IL-9, basic fibroblast growth factor (b-FGF), granulocyte-colony stimulating factor (GCSF), granulocyte-macrophage colony-stimulating factor (GM-CSF), interferon-gamma (IFNγ), inducible protein 10 (IP10), monocyte chemoattractant protein-1 (MCP-1), macrophage inflammatory protein-1A (MIP1A) and MIP1B, platelet-derived growth factor (PDGF), tumor necrosis factor-alpha (TNFα), and vascular endothelial growth factor (VEGF) in their serum levels. When serum levels of ICU-patients were compared to non-ICU patients, IL-2, IL-7, IL-10, GCSF, IP10, MCP1, MIP1A, and TNFα were elevated in ICU patients (Channappanavar and Perlman, 2017). Furthermore, patients who develop mild or high acute respiratory syndrome due to SARS-CoV-2 infection show an increased level of IL-1β and IL-6, which mediate lung inflammation, fever, and fibrosis (Gallagher and Buchmeier, 2001). It has been reported that IL-6 is one of the main cytokines involved in pulmonary complications associated with SARS-CoV-2 infection (Simmons et al., 2005). Therefore, the inhibition of these overexpressed cytokines could be a potential therapeutic target for COVID-19. Numerous potential therapeutic targets associated with coronavirus infections in humans have been identified (**Table 2**).

**Table 2:** Potential therapeutic targets associated with coronavirus infections in humans.

Target	Function	Coronavirus type	Reference
Angiotensin-converting enzyme 2 (ACE2)	Functional cellular receptor for SARS-CoV and SARS-CoV-2 (COVID-19)^*^	SARS-CoV SARS-CoV-2	(Yan et al., 2020)
Spike glycoprotein (S protein)—during viral infection in cleaved into S1 and S2 subunits	Mediates receptor recognition and membrane fusion for viral entry. S1 subunit: contains receptor-binding domain (RBD)^**^ which binds to the peptidase domain (PD) of ACE2 S2 subunit: responsible for membrane fusion; cleaved by host proteases once S1 binds to ACE2 which is needed for a viral infection to occur	SARS-CoV	(Gallagher and Buchmeier, 2001)
Cathepsin L–cysteine peptidase	Facilitates the cleavage of the S protein of SARS-CoV, therefore aids in the activation of membrane fusion	SARS-CoV	(Simmons et al., 2005)
Transmembrane protease serine 2 (TMPRSS2)	Cleaves C-terminal segment of ACE2, enhancing S-protein viral infection	SARS-CoV	(Shulla et al., 2011)
Nonstructural protein 1 (Nsp1) coronavirus virulence factor	Induces host mRNA degradation by interacting with the hosts 40S ribosomal subunit and inhibits type-I interferon production	SARS-CoV	(Kamitani et al., 2006; Narayanan et al., 2008)
Open reading frame 7a (ORF7a) coronavirus virulence factor	ORF7a binds directly to bone marrow stromal antigen 2 (BST-2), blocking the activity of BST-2 by disrupting the glycosylation of BST-2. BST-2 mediates the restriction of virus-like particle release	SARS-CoV	(Taylor et al., 2015)
Replicase polyproteins	Involved in the transcription and replication of viral RNAs. Encoded by open reading frames (ORF) 1a and 1b.	SARS-CoV	(Wu et al., 2020)
Papain-like proteinase (PL^pro^)	Essential in the replication and infection for coronaviruses. Cleaves the N-terminal of the replicase polyprotein causing the release of Nsp1, Nsp2 and Nsp3, which are in turn involved in viral replication	SARS-CoV	(Harcourt et al., 2004)
Viral main protease (3CL^pro^, also called Mpro) –cysteine protease	Controls the activities of the coronavirus replication complex and is therefore essential for viral replication^***^	SARS-CoV SARS-CoV-2	(Anand et al., 2003)
RNA dependent RNA polymerase (RdRp) (nsp12)	Essential protease enzyme that catalyzes the replication of RNA from the RNA template	SARS-CoV SARS CoV-2	(Lung et al., 2020)
Non-structural protein 13 (NSP13)/helicase	Enhances the efficiency of viral replication and proliferation through its NTPase, duplex RNA/DNA-unwinding and RNA-capping activities	SARS CoV	(Shum and Tanner, 2008)

^*^Functional cellular receptor (ACE2) are identical for SARS-CoV and SARS-CoV-2 (Ou et al., 2020); ^**^Homology of the spike-receptor binding domain (RBD) sequence between SARS-CoV-2 and SARS-CoV is 76% (Wu et al., 2020); ^***^The SARS-CoV-2 main protease is closely related (96% identity) to the SARS-CoV protease (Chen and Du, 2020).

COVID-19 infections in humans are not only associated with various pulmonary complications or respiratory illnesses but also several other organs, such as the kidney and liver, are also affected, which could contribute toward impaired metabolism and excretion of potential drugs used to treat the disease (Rismanbaf and Zarei, 2020). A study by Zhang et al. (2020a), reported that 2–11% of patients infected with COVID-19 showed signs of liver dysfunction with 14–53% of cases displaying elevated levels of alanine aminotransferase (ALT) and aspartate aminotransferase (AST). This was confirmed in a study by Huang et al. (2020), where increased levels of AST were detected in 37% of COVID-19 patients (Huang et al., 2020). Guan et al. (2020) found that elevated AST and ALT levels were more prominent in patients with severe COVID-19 compared to non-severe cases. Additionally, this study reported that, on admission, 83.2% of patients suffered from lymphocytopenia (low levels of lymphocytes in the blood), 36.2% had thrombocytopenia (low blood platelet count), and 33.7% had leukopenia (low white blood cell count) (Guan et al., 2020). In a study by Xu et al. (2020), biopsies were taken from the lung, liver, and heart tissue of a patient who died from a cardiac arrest associated with COVID-19. Histological examination of the liver tissue revealed that the patient showed moderate microvesicular steatosis and mild lobular and portal activity, which could have been due to the COVID-19 infection or drug-induced damage, whereas a few interstitial mononuclear inflammatory infiltrates were found in the heart tissue (Xu et al., 2020). Additionally, several features characteristic of COVID-19 infection were found within the lung tissue, such as pulmonary oedema and alveolar damage (Xu et al., 2020). Acute kidney injury has also been reported as a severe symptom in patients hospitalized with COVID-19. Hirsch et al. (2020) reported that 36.6% of patients admitted with COVID-19 developed acute kidney injury, which was most prominent in patients with respiratory failure (89.7% of patients on ventilators compared to 21.7% not using ventilators) (Hirsch et al., 2020). The expression of ACE2 receptors are not only prevalent in lung cells but are also expressed in kidney cells; however, it has been reported that the incidence of acute kidney injury (29%) is lower than incidence of lung damage (71%) associated with COVID-19 infection (Malha et al., 2020).

A study by Zou et al. (2020) aimed at identifying high-risk organs vulnerable to COVID-19 infection through single-cell RNA sequencing techniques. This study identified the lungs, heart, bladder, kidneys, oesophagus, and ileum as high-risk organs for COVID-19 infection, specifically identifying type II alveolar lung cells, myocardial cells, bladder urothelial, ileum, oesophagus epithelial, and kidney proximal tubule cells, which express ACE2 (Zou et al., 2020). Xiao et al. (2020) found that 53.42% of COVID-19 hospitalized patients tested positive for SARS-COV-2 RNA in stool samples, of which 23.29% tested negative for SARS-CoV-2 when respiratory samples were tested, which confirms that SARS-CoV-2 is able to infect the gastrointestinal system, which also suggests that the spread of COVID-19 could be through fecal-oral transmission (Xiao et al., 2020). The infection of the gastrointestinal tract could furthermore explain the prevalence of diarrhea in COVID-19 patients, which highlights the need to monitor individuals with diarrhea as a potential initial symptom of COVID-19 infection (Zhang et al., 2020d).

A study by Varga et al. (2020) described the involvement of vascular endothelial cells, which express ACE2 receptors, in multi-organ toxicity related to COVID-19 infected patients. Histological analysis of a patients’ tissue, who suffered from pre-existing heart conditions, showed that there was an increase in inflammatory cells associated with the endothelium and an increase of mononuclear cells in the lung, as well as the presence of apoptotic bodies in the heart, lung, and small bowel. Histological analysis of a second patients’ tissue, who suffered from heart comorbidities and obesity, showed the presence of lymphocytic endotheliitis in the lung, heart, kidney and liver; necrosis of liver cells; and endotheliitis of the submucosal vessels in the small intestine. In a third patient who suffered from high blood pressure, endotheliitis of the submucosal vessels in the small intestine was also observed and the presence of apoptotic bodies. Varga and colleagues were able to conclude that SARS-CoV-2 is able to directly infect endothelial cells, thereby causing endotheliitis in several organs and increased inflammatory response (Varga et al., 2020).

Although SARS-CoV and SARS-CoV-2 share several similarities, there are many differences. SARS-CoV-2 is considered the most contagious, as asymptomatic hosts can spread the virus *via* respiratory droplets and contaminated fomites (Chen et al., 2020; Lai et al., 2020; Prompetchara et al., 2020; Yuen et al., 2020), whereas SARS-CoV can only be spread by those that have severe respiratory illnesses (Lung et al., 2020; Wilder-Smith et al., 2020). This has allowed SARS-CoV-2 to infect more countries and have higher case numbers than SARS-CoV and MERS-CoV (Arabi et al., 2020; Wu et al., 2020). Numerous studies have been conducted on the use of medicinal plants and their isolated secondary metabolites to target and inhibit proteins related to coronavirus infections in humans. Following the outbreak of SARS in China during 2002, the State Administration of Traditional Chinese Medicine of the People’s Republic of China initiated clinical research projects regarding the combined use of Traditional Chinese medicine (TCM) and Western medicine for treating SARS. A total of 21 research projects were initiated to cover three aspects of SARS, namely, prevention, treatment, and rehabilitation (World Health Organization, 2004).

Of the 5327 patients diagnosed with SARS across the country, 3104 cases received TCM treatment. The WHO reviewed clinical and research reports on patients treated with a combination of Traditional Chinese Medicine and Western Medicine to better understand the potential of these treatments for SARS. They concluded that the integrated use of TCM and Western medicine for SARS patients was safe and that there could be potential benefits to SARS patients using this combined treatment method. A reduction in case fatality rate, when treated with the combination therapy as opposed to treatment with Western medicine alone, was also observed. In addition to these benefits, the combination treatment regime lowered the overall cost of effective treatment. This highlights the importance of introducing complementary medicine, such as through the use of medicinal plants, for the treatment of SARS (World Health Organization, 2004).

## Literature Study on the Use of Natural Products Against Coronaviruses

**Figure 2:**
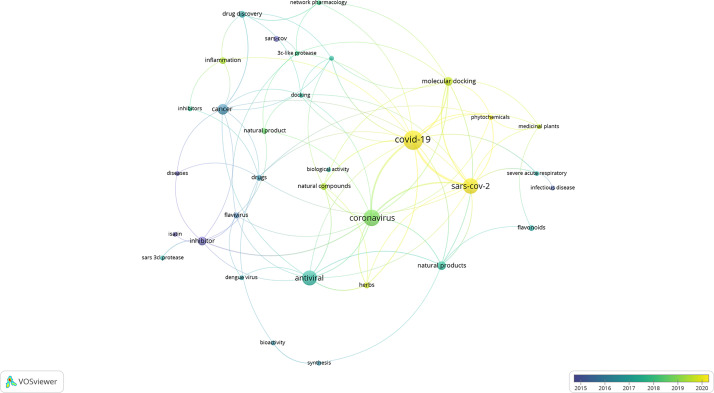
Network map of the literature data analysis (2010 to 2020). Circles represent identified keywords and the size correspond to the occurrence count of the keyword. Curved lines represent the connectivity between different keywords. The color corresponds to the year associated with the specific keyword.

To assess the current literature on the potential use of natural products against coronaviruses, a detailed literature study was conducted using published research articles ranging from the year 2010–2020. This analysis was conducted to indicate the current state of the art and identify potential gaps and areas in the field that can be explored in future research studies. Four databases were used to conduct the literature search, namely, ScienceDirect, SciFinder^n^, Scopus, and Web of Science. The search terms included “coronavirus” and “natural product*.” VOSviewer was used to analyse the co-occurrence of related keywords. Similar trends and keywords were identified in each of the databases. ScienceDirect, followed by Scifinder^n^, identified the largest hit ratio with 120 and 124 papers, respectively. The most recent, prevailing, and obvious co-occurrence of keywords were “SARS-CoV-2” and “COVID-19” (**Figure 2**). This was followed by the identification of the keywords “medicinal plants,” “natural products,” “natural compounds,” and “phytochemicals.” The only potential drug target or mechanism that was identified, and associated with natural products, was the cysteine protease, 3CL^pro^. Similarly, flavonoids were identified as the largest class of compounds with potential activity; however, this group does not have any link to the 3CL^pro^ group, indicating that the mechanism is poorly understood and has not yet been identified. Research articles which include computational approaches, such as molecular docking, have increased over the past few months, which is expected due to the rapid outcome of results using these approaches. Molecular docking and its applicability to identifying potentially biologically active compounds are later discussed in this review. Due to the outbreak of SARS-CoV-2 as a new and emerging disease, it is expected that the body of published research is still fairly limited, and it is of utmost importance to structure future research projects with a clear hypothesis, research justification, and relevant and appropriate methods.

**Figure 3:**
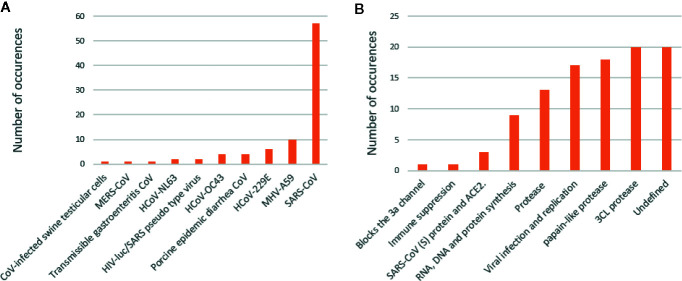
Test systems used in the assessment of natural products against coronaviruses **(A)**. Proposed mechanisms associated with natural products **(B)**.

Analysis of the test systems used, as well as the proposed mechanisms associated with natural products activity, revealed interesting trends (**Figure 3**). The data has been compiled from Islam et al. (2020). The most prevalent test system used to date is the SARS-CoV-1, regardless of the strain. Moreover, there are some reports on other coronavirus strains, including MERS-CoV and coronaviruses associated with other animal diseases. Many of the proposed mechanisms were ‘undefined,’ indicating one of the major concerns and obstacles in drug discovery and natural product pharmacology. The proteases, 3CL^pro^ and PL^pro^, were identified as the second and third most investigated proposed mechanisms associated with natural product activity, respectively. The group, ‘viral infection and replication,’ was identified as the fourth-highest proposed mechanism. The exact molecular targets have not been identified in these reports; however, it can be hypothesized that inhibition of viral infection can be associated with the ACE2 receptor. The success of natural product research as anti-coronavirus compounds does not only lie in the rapid identification of active compounds but also the identification of a targeted mechanism of action (Islam et al., 2020).

## Plants and Isolated Compounds With Activity Against SARS-CoV Targets and Other Human Coronaviruses

**Figure 4:**
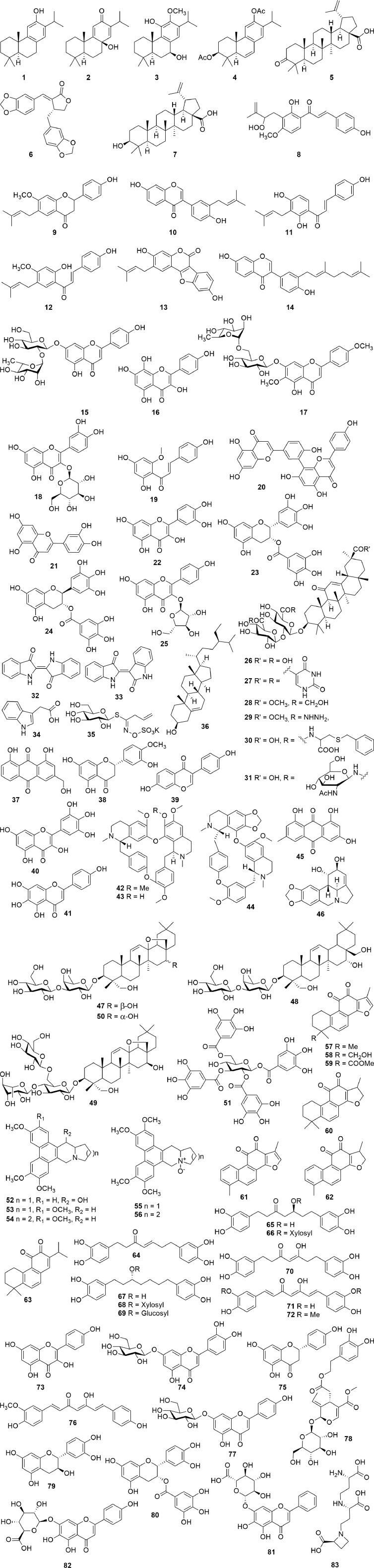
Chemical structures, which target proteins associated with SARS-CoV (1–17).

Several natural products have shown activity and their structures are represented in **Figure 4**. A study conducted by Wen et al. (2007) investigated whether 22 terpenoids and lignoids were able to inhibit viral replication of SARS-CoV in African green monkey kidney (Vero) E6 cells. The cytotoxic effect of the compounds against Vero E6 cells and the ability to inhibit viral replication were measured. The most potent compounds were found to be ferruginol (**1**), 8*β*-hydroxyabieta-9(11),13-dien-12-one (**2**), 7*β*-hydroxydeoxycryptojaponol (**3**), 3*β*,12-diacetoxyabieta-6,8,11,13-tetraene (**4**), betulonic acid (**5**), and savinin (**6**). Compounds **1**–**6** were found to be potent inhibitors of viral replication with effective concentrations (EC_50_), concentration where 50% of viral replication was inhibited, of 1.39, 1.47, 1.15, 1.57, 0.63, and 1.13 µM, respectively (Wen et al., 2007).

The selective index (SI) values of compounds **1**–**6** were found to be 58, >510, 111, 193, 180, and >667, respectively, indicating that these plants were able to inhibit viral replication without having a cytotoxic effect on the host cells. Compounds **1**, **2**, and **6** were purified from the ethyl acetate extracts of the heartwood of *Chamaecyparis obtuse* var. *formosana* Hayata, whereas compounds **4** and **5** were isolated from the heartwood of *Juniperus formosana* Hayata and compound **3** from *Cryptomeria japonica* (Thunb. ex L.f.) D.Do. Furthermore, betulinic acid (**7**) and savinin (**6**) were able to inhibit SARS-CoV 3CL protease activity (3CL^pro^) with IC_50_ of 10 and 25 µM. The inhibitory mechanism of betulinic acid (**7**) and savinin (**6**) was also calculated, showing Ki values of 8.2 ± 0.7 and 9.1 ± 2.4 µM, respectively, with a competitive mode of inhibition (Wen et al., 2007).

A chalcone, xanthoangelol E (**8**), isolated from the ethanolic leaf extract of *Angelica keiskei* (Miq.) Koidz., showed inhibitory activity against SARS-CoV 3CL^pro^ and a papain-like protease (PL^pro^) with IC_50_ values of 11.4 and 1.2 µM, respectively, using cell-free assays. The chalcone was shown to be a competitive inhibitor of the SARS-CoV 3CL^pro^, whereas noncompetitive inhibition was observed with the SARS-CoV PL^pro^. In a cell-based assay, xanthoangelol E (**8**) showed an IC_50_ value of 7.1 µM against the SARS-CoV 3CL^pro^ and a 50% cytotoxic concentration (CC_50_) of 65.6 µM against Vero cells (SI = 9.2) (Park et al., 2016). In a study by Kim et al. (2014), six flavonoid compounds bavachinin (**9**), neobavaisoflavone (**10**), isobavachalcone (**11**), 4’-*O*-methylbavachalcone (**12**), psoralidin (**13**), and corylifol A (**14**) were isolated from the ethanolic extract of the seeds of *Psoralea corylifolia* L. Each of these compounds were able to inhibit PL^pro^ in a dose-dependent manner. Compounds **11** and **13** showed the highest inhibition with IC_50_ values of 7.3 and 4.2 µM, respectively, whereas the other compounds showed lower inhibition with IC_50_ values ranging between 10.1 and 38.4 µM (Kim et al., 2014).

In a recent study by Jo et al. (2020), a flavonoid library was used to examine whether these compounds displayed inhibitory activity against SARS-CoV 3CL^pro^. The compounds rhoifolin (**15**), herbacetin (**16**), and pectolinarin (**17**) were found to have noteworthy inhibitory activity against 3CL^pro^ with IC_50_ values of 27.45, 33.17, and 37.78 µM, respectively (Jo et al., 2020). The authors, furthermore reported that the compounds herbacetin (**16**), isobavachalcone (**11**), quercetin-3-*β*-D-glucoside (**18**), and helichrysetin (**19**) were able to inhibit MERS-CoV 3CL^pro^ with IC_50_ values of 40.59, 35.85, 37.03, and 67.04 µM, respectively (Jo et al., 2019). In another study, it has been estimated, through a bioinformatic meta-analysis, that the leaves of the Barley varieties, Stratus, and Morex, as well as the leaves of *Ficus deltoidea* Jack, contain high percentages of rhoifolin (**15**), while the leaves of *Cirsium chlorolepis* Petr. ex Hand.-Mazz. contain a high quantity of pectolinarin (**17**). The authors, therefore, hypothesize that these plants may be effective in inhibiting coronaviruses; however, the plant extracts have not been tested (Sawikowska, 2020).

A study by Wen et al. (2011) reported that an ethanolic rhizome extract of *Cibotium barometz* (L.) J.Sm., a hexane rhizome extract of *Gentiana scabra* Bunge, a methanolic tuber extract of *Dioscorea batatas* Decne., a hexane seed extract of *Cassia tora* L. and a hexane stem and leaf extract of *Taxillus chinensis* (DC.) Danser showed effective inhibition of SARS-CoV replication in Vero E6 cells with EC_50_ values of 8.42, 8.70, 8.06, 8.43, and 5.39 µg/mL, respectively. The SI values were found to be >59.4, >57.5, >62.0, >59.3, and >92.8, respectively, with each of the extracts having a CC_50_ value of >500 µg/mL. Additionally, the methanolic extracts of *C. barometz* and *D. batatas* showed inhibition of SARS-CoV 3CL^pro^ with IC_50_ values of 39 and 44 μg/mL (Wen et al., 2011).

The essential oil from *Laurus nobilis* L. exhibited inhibition against SARS-CoV with an EC_50_ value of 120 µg/mL and an SI value of 4.16 (Loizzo et al., 2008). At a concentration of 100 µg/mL, an ethanolic leaf extract of *Torreya nucifera* (L.) Siebold and Zucc. exhibited 62% inhibition of SARS-CoV 3CL^pro^ compared to the untreated enzyme control. Through bioassay-guided fractionation, the compound amemtoflavone (**20**), a biflavone, was isolated, which showed the most potent 3CL^pro^ activity with a non-competitive IC_50_ value of 8.3 µM. In this study, luteolin (**21**) and quercetin (**22**) were also tested, which showed IC_50_ values of 20.2 and 23.8 µM, respectively. The type of inhibition for these two compounds could not be determined, which might be indicative of a false positive (Ryu et al., 2010). In a study by Nguyen et al. (2012), quercetin (**22**) was reported to have an IC_50_ value of 73 µM against SARS-CoV 3CL^pro^; however, no mention is made to the type of enzymatic inhibition. Luteolin (**21**) and quercetin (**22**) are known as pan-assay interference compounds due to the catechol moiety. Additional assays are required if a compound is classified as a pan assay interference compound (PAINS) (Nguyen et al., 2012). To determine specific enzyme activity, the assays should include counter-screening on unrelated targets, kinetic investigation to determine if the compound is a competitive or non-competitive inhibitor, and clearly identifying and carefully describing the concentration-response curves (Aldrich et al., 2017). Both epigallocatechin gallate (**23**) and gallocatechin gallate (**24**) also inhibited SARS-CoV 3CL^pro^ with IC_50_ values of 73 and 47 µM, respectively. Furthermore, gallocatechin gallate (**24**) was found to be a competitive inhibitor of 3CL^pro^ with a Ki value of 25 µM (Nguyen et al., 2012). Similar to luteolin (**21**) and quercetin (**22**), both epigallocatechin gallate (**23**) and gallocetechin gallate (**24**) contain substructures classified as PAINS. To further assess the antiviral activity, PAINS need to undergo cellular-based inhibitory assays in order to eliminate false positives. The different cell-based assays are briefly summarized in the discussion section. The compound juglanin (**25**), a glycoside of kaempferol, was shown to effectively inhibit the 3a-mediated current with an IC_50_ of 2.3 μM. The protein which is encoded by the open-reading frame 3a (ORF3a) of SARS is involved in virus release and production (Schwarz et al., 2014). Glycyrrhizin (**26**), a triterpenoid glycoside isolated from *Glycyrrhiza glabra* L., was one of the first compounds found to inhibit SARS-CoV replication *in vitro*. Several derivatives (**27**–**31**) of glycyrrhizin (**26**) have also been synthesized which showed up to 70-fold increased activity. Glycyrrhizin (**26**) was found to inhibit viral replication with an EC_50_ value of 365 μM and an SI value of >65. The EC_50_ values for derivatives **27**–**31** were 5.0, 8.0, 16.0, 35.0, and 40.0 μM, respectively, while the SI values were 3, 6, 4, 41, and >75, respectively (Hoever et al., 2005). In a similar study, **26** was found to inhibit the cytopathic effect of SARS-CoV with an EC_50_ value of 300 µg/mL and an SI value of >33. (Cinatl et al., 2003).

A root extract of *Isatis indigotica* Fortune ex Lindl., as well as compounds isolated from the plant; indigo (**32**), indirubin (**33**), indican (**34**), sinigrin (**35**), *β*-sitosterol (**36**), aloeemodin (**37**), hesperetin (**38**), and daidzein (**39**) were able to inhibit the cleavage of 3CL^pro^ in a cell-free assay with IC_50_ values of 53.8, 37.3, 81.3, 33.1, 50.3, 47.8, 35.7, 18.1, and 26.8 µg/mL, respectively (Lin et al., 2005). However, when tested in a cell-based assay, only the extract and indigo (**32**), sinigrin (**35**), *β*-sitosterol (**36**), aloeemodin (**37**), and hesperetin (**38**) showed inhibition, with IC_50_ values of 191.6, 190, 90.1, 502.1, 99.1, and 2.5 µg/mL, respectively (Lin et al., 2005). An aqueous extract prepared from the whole plant of *Houttuynia cordata* Thunb. showed low inhibition of both SARS 3CL^pro^ and RdRp activity in a dose-dependent manner with 50% inhibition of 3CL^pro^ at a concentration >1,000 µg/mL and 50% inhibition of RdRp activity at >200 µg/mL. Although a concentration-response curve was present in the reported activity, the inhibitory activity, on both 3CL^pro^ and RdRp, is lower when compared to other plants. The study does not report on the potential active compound/s, and therefore, bioassay-guided fractionation is needed to identify bioactive compounds. However, this is unlikely due to the low activity observed. Moreover, the study reported acute oral toxicity conducted in mice, which found that the extract was non-toxic when administered at 16 g/kg (16,000 mg/kg). However, there was a mortality rate of 10% among the female mice (Lau et al., 2008). In addition, this dosage is considered extremely high, exceeding the recommended upper limit of 2000 mg/kg (5,000 mg/kg in extreme cases), set out by the OECD guidelines (Erhirhie et al., 2018). In addition, the study by Lau et al. (2008) identified that the extract was able to induce T cell proliferation, specifically CD4^+^ and CD8^+^ T cells in an *in vitro* splenic lymphocyte assay at concentrations ranging from 50–400 µg/mL (Lau et al., 2008). Woranam et al. (2020) reported that aqueous and methanolic extracts prepared from the aerial parts of *H. cordata*, at concentrations ranging between 5–750 and 4–12 µg/mL, respectively, were able to reduce nitric oxide production in murine macrophages (RAW 264.7) and decreased the expression of PGE2, iNOS, IL-1β, TNF-α, and IL-6 in LPS-stimulated RAW 264.7 cells (Woranam et al., 2020). Therefore, this plant should rather be considered as a potential immune modulator, as opposed to an antiviral, but further investigation is needed.

In a study by Yu et al. (2012), 64 compounds were tested for their inhibitory activity against the SARS helicase enzyme (nsP13). Myricetin (**40**) and scutellarein (**41**) were able to significantly inhibit nsp13 ATPase activity with IC_50_ values of 2.71 and 0.83 µM, respectively. Furthermore, cytotoxicity studies revealed that these compounds, at a concentration of 2 µM, did not affect the growth of normal epithelial breast cells (MCF10A) (Yu et al., 2012).

The alkaloids tetrandrine (**42**), fangchinoline (**43**), and cepharanthine (**44**) were able to inhibit the cytopathic effect of HCoV-OC43 in human lung cells (MRC-5) with EC_50_ values of 295.6, 919.2, and 729.7 nM, respectively. The cytotoxic effect of the compounds in the MRC-5 cells was determined and showed CC_50_ values of 15.51, 12.40, and 10.54 µM and SI values of >40, 11, and 13, respectively (Kim et al., 2019). These compounds additionally were able to inhibit the expression levels of the N and S proteins and the inflammatory cytokines interleukin 1β (IL-1β), IL-6, and IL-8. Furthermore, Zou et al. (2019) reported that tetrandrine (**42**) was able to inhibit pro-inflammatory Th1, Th2, and Th17 cells (Zou et al., 2019).

A chloroform fraction, from an ethyl acetate partition, from a 75% ethanolic extract of the whole plant of *Rheum palmatum* L., showed a high inhibition of SARS-CoV 3CL^pro^ with an IC_50_ value of 13.76 µg/mL. The inhibitory activity of the crude extract was 38.09 µg/mL, while the fractions and partitions showed inhibition ranging between 13.76 and 59.33 µg/mL (Luo et al., 2009). The study does not report the identification of an active compound or the mechanism of action by binding to a specific target. In addition, the activity does not appear to be specific to polarity, which may be indicative of a false positive. An in-depth investigation is needed to confirm the suitability of *R. palmatum* and its constituents as potential candidates for further investigation. A water extract prepared by boiling (decoction) the leaves of *Toona sinensis* (Juss.) M. Roem. inhibited HCoV 229E viral replication in Vero cells with an EC_50_ of 30 µg/mL. This plant is consumed as a cooked vegetable and the extract was, therefore, prepared from the cooked/boiled menstruum. When the extract preparation did not include boiling, an EC_50_ value of 43 µg/mL was obtained. Both the boiled and non-boiled extract did not show cytotoxic effects against the Vero cells, with CC_50_ values of >500 µg/mL and SI values of 17 and >12, respectively. The proposed active compound/s, mechanism of action, and the effect of boiling on the chemical profile have not been identified or discussed (Chen et al., 2008). Although the article identified a difference in activity between boiled and non-boiled extracts, the difference appears insignificant, however, the difference noted in the activity might be due to the breakdown and release of glucose and an aglycon from glycosides during the heating process (Fabbri and Chiavari, 2001). The compound emodin (**45**), found within the genus *Rheum* and *Polygonum*, was able to block the binding of the SARS-CoV S protein to the ACE2 receptor with an IC_50_ value of 200 µM (Ho et al., 2007). Emodin (**45**) was furthermore able to inhibit the SARS-CoV and HCOV-OC43 3a ion channel with a K_1/2_ value of 20 µM (Schwarz et al., 2011). An ethanolic stem cortex extract of *Lycoris radiata* (L’Hér.) Herb. exhibited anti-SARS-CoV activity against viral strains BJ-001 and BJ-006 with EC_50_ values of 2.4 and 2.1 µg/mL and SI values of 370 and 422, respectively. The EC_50_ and SI values for the total alkaloid fraction from *L. radiata* was found to be 1.0 µg/mL and 94, respectively. This led to the isolation of lycorine (**46**) from *L. radiate*, which showed significant inhibition with an EC_50_ and SI value of 15.7 µg/mL and 954, respectively (Li et al., 2005).

Saikosaponins, which are oleanane derivatives, were tested for antiviral activity against the coronavirus 229E. Saikosaponin A (**47**), B_2_ (**48**), C (**49**), and D (**50**) showed inhibition of HCoV-229E viral infection in MRC-5 cells with EC_50_ values of 8.6, 1.7, 19.9, and 13.2 µmol/L. These saikosapnins furthermore were not cytotoxic to the MRC-5 cells with CC_50_ values of 228.1, 383.3, 151.5, and 176.2 µmol/L with SI values of 26.6, 221.9, 19.2, and 13.3, respectively. In addition, saikosaponin B_2_, which showed the highest activity, was able to inhibit viral attachment and penetration (Cheng et al., 2006). In a study by Yi et al. (2004), tetra-*O*-galloyl-*β*-D-glucose (**51**) and luteolin (**21**) were tested for their activity against SARS-CoV. Both compounds were able to dose-dependently inhibited SARS-CoV infection in Vero E6 cells with EC_50_ values of 4.5 and 10.6 µM, respectively. The cytotoxic effect was also determined, and both were found to be non-toxic with CC_50_ values of 1.08 and 0.115 mM and SI values of 240 and 14.62, respectively (Yi et al., 2004).

Tylophorinine (**52**), isolated from *Tylophora indica* (Burm. f.) Merr., and four synthetic an tylophorine (**53**), 7-methoxycryptopleurine (**54**), tylophorine N-oxide (**55**) (a naturally occurring compound), and 7-methoxycryptopleurine N-oxide (**56**) showed significant activity against SARS-CoV with EC_50_ values ranging from <5 to 340 nM and SI values ranging from 1.7 to >100 (Yang et al., 2010). Tanshinones (**57**–**63**), isolated from *Salvia miltiorrhiza* Bunge, were found to be time-dependent selective inhibitors against the cysteine protease SARS-CoV PL^pro^. Tanshinone IIA (**57**), tanshinone IIB (**58**), methyl tanshinonate (**59**), cryptotanshinone (**60**), tanshinone I (**61**), and dihydrotanshinone I (**62**) were identified as non-competitive enzyme isomerization inhibitors, whereas rosmariquinone (**63**) showed a mixed-type simple reversible slow-binding inhibition. The IC_50_ values of compounds **57**–**63** were found to range between 0.8 and 30.0 µM against SARS-CoV PL^pro^ and between 14.4 and 226.7 µM against SARS-CoV CL^pro^ (Park et al., 2012b). Six diarylheptanoids (**64**–**69**), isolated from *Alnus japonica* (Thunb.) Steud., as well as two synthetic derivatives (**70**–**71**), showed inhibitory activity against SARS-CoV PL^pro^ with IC_50_ values ranging between 4.1 and 59.8 µM. Curcumin (**72**) was used as a positive control in this study, which showed an IC_50_ value of 5.7 µM (Park et al., 2012a).

## Prospects of Using Computational Techniques to Screen Possible Anti-COVID-19 Agents From Plants

Zhang et al. (2020b), recently reported the crystal structure of the SARS-CoV-2 main protease (Mpro also called 3CL^pro^), which is essential for viral replication. The availability of the crystal structure allows compounds, which have shown activity against SARS-CoV proteases, and other similar compounds to be screened through computational studies to identify possible lead molecules active against COVID-19. Based on a molecular docking study reported by Khaerunnisa et al. (2020), kaempferol (**73**), quercetin (**22**), luteolin-7-*O*-glucoside (**74**), naringenin (**75**), desmethoxycurcumin (**76**), curcumin (**72**), apigenin-7-*O*-glucoside (**77**), oleuropein (**78**), catechin (**79**), and epicatechin-gallate (**80**) could potentially inhibit SARS-CoV-2 3CL^Pro^ and therefore act as anti-COVID-19 agents (**Figure 4**); however, *in vitro* studies are required to assess these results further (Khaerunnisa et al., 2020). According to another report, the host receptor for SARS-CoV-2, ACE2, is the same as the host receptor of SARS-CoV; therefore, the inhibitors of SARS-CoV ACE2 might be able to inhibit the same receptor in SARS-CoV-2 (Salata et al., 2019). Based on the molecular docking study performed by Chen and Du (2020), baicalin (**81**), scutellarin (**82**), hesperetin (**38**), nicotianamine (**83**), and glycyrrhizin (**26**) have been identified as potential ACE2 inhibitors and could be used as possible anti-2019-nCoV agents (Chen and Du, 2020). Molecular docking can be a useful tool to describe binding affinities and molecular interactions and is a rapid technique in which to identify potentially active compounds during drug discovery. However, *in vitro* or *in vivo* antiviral tests are crucial in order to support molecular docking data, which describes a compound with potent activity. Studies have shown that a positive correlation between docking scores and pharmacological activity are relatively low and docking is not very effective in ranking active compounds (Vilar and Costanzi, 2012). This emphasizes the need to include wet-lab experimentation to substantiate the activities of natural products, especially in the context of a global pandemic.

## Potential Leads From Southern African Plants

In Southern Africa, a major portion of the population relies primarily on traditional medicine as a source of health care. In traditional knowledge systems, the use of a plant for the treatment of a specific symptom, rather than a specific disease or infectious organism is recorded. In this section, Southern African plants that are traditionally used in the treatment of coughs, fevers, colds, and influenza have been listed as potential candidates for testing against SARS-CoV-2 and related targets (**Table 3**) (Van Wyk et al., 2009). This aids in identifying a large number of potential plant species, especially Southern African plants, which can be considered for investigating the potential inhibition against coronaviruses. Only a few of the plant species listed in **Table 3** have been tested for their antiviral potential, indicating the major gap in scientifically assessing the medicinal potential of traditionally used plants, thereby emphasizing the importance for African-based researchers to include these types of studies within their research focus. Furthermore, extensive toxicity and *in vivo* testing is necessary to investigate the pharmacological use of these plants and compounds.

**Table 3:** Potential Southern African medicinal plants (traditionally used for coughs, fevers, colds and influenza) that showed activity against coronaviruses or against similar viruses [the list have been compiled from Medicinal Plant of South Africa (Van Wyk et al., 2009)].

Name	Vernacular name	Reported activity against human coronaviruses
*Adansonia digitata* L.	Kremetart, Baobab, Shimuwu, Movana, Muvhuyu	NT^#^
*Agathosma betulina* (P.J.Bergius) Pillans	Boegoe, Buchu, Ibuchu	NT
*Alepidea amatymbica* Eckl. & Zeyh.	Kalmoes, Lesoko, Iqwili, Ikhathazo	NT
*Aloe excelsa* A.Berger	Noble aloe, Zimbabwe aloe	NT
*Artemisia afra* Jacq. ex Willd.	Als, Wildeals, African wormwood, Lengana, Umhlonyane	*Artemisia annua*, closely related species to *A. afra*: EC_50_^+^ = 34.5 ± 2.6 μg/mL (SARS-CoV BJ-001); CC_50_^++^ = 1053 ± 92.8 μg/mL (Vero cells); SI^##^ = 27 (Li et al., 2005)
*Aspalathus linearis* (Burm.f.) R.Dahlgren	Rooibostee, Rooibos tea	Quercetin: IC_50_^+++^ = 73 μM (Recombinant 3CL^pro^) (Nguyen et al., 2012)
		Luteolin: EC_50_ = 10.6 μM (wild-type SARS-CoV); CC_50_ = 0.16 mM (Vero cells); SI = 14.62 (Yi et al., 2004)
*Ballota africana* (L.) Benth.	Kattekruid	NT
*Camellia sinensis* (L.) Kuntze	White tea, green tea, mchai (Kiswahili)	Epigallocatechin gallate: IC_50_ = 73 μM (Recombinant 3CL^pro^) (Nguyen et al., 2012)
*Cannabis sativa* L.	Dagga, Marijuana, Matokwane, Umya, Nsangu	NT
*Catha edulis* (Vahl) Endl.	Boesmanstee, Khat, Bushman’s tea	NT
*Chondropetalum mucronatum* (Nees) Pillans	Mountain Restio	Myricetin: IC_50_ = 2.71 ± 0.19 μM (nsP13, SARS helicase protein); Cytotoxicity: No toxicity at 2 μM against MCF10A cells (Yu et al., 2012)
		Quercetin: IC_50_ = 73 μM (Recombinant 3CL^pro^) (Nguyen et al., 2012)
*Cinnamomum camphora* (L.) J.Presl	Kamferboom, Camphor tree, Uroselina	NT
*Croton gratissimus* Burch.	Bergboegoe, Lavender croton, Maquassie, Umahlabekufeni	NT
*Cyclopia latifolia* DC.	Heuningbos, Honeybush	Epigallocatechin gallate: IC_50_ = 73 μM (Recombinant 3CL^pro^) (Nguyen et al., 2012)
		Luteolin: EC_50_ = 10.6 μM (wild-type SARS-CoV); CC_50_ = 0.16 mM (Vero cells); SI = 14.62 (Yi et al., 2004)
*Datura stramonium* L.	Stinkblaar, Thornapple, Lethsowe, Zaba-zaba, Iloyi, Ijoyi	NT
*Dicoma capensis* Less.	Wilde karmedik, Koorsbossie	NT
*Dodonaea viscosa* (L.) Jacq.	Sandolien, Sand olive, Mutepipuma, Mutata-vhana	β-sitosterol: EC_50_ = 1210 μM (HCoV-NL63) (Lin et al., 2005)
*Drimia elata* Jacq.	Brandui, Indongana-zibomvana	NT
*Glycyrrhiza glabra* L.	Soethoutwortel, Liqourice root, Mlomo-mnandi	Glycyrrhizin: EC_50_ = 300 mg/L (SARS-CoV); CC_50_ >20 000 mg/L (Vero cells); SI >67 (Cinatl et al., 2003)
*Halleria lucida* L.	Tree fuschia, white olive	NT
*Helichrysum* spp.	Kooigoed, Everlastings, Isicwe, Imphepho	Helichrysetin: IC_50_ = 67.04 μM (MERS‐CoV 3C like-protease) (Jo et al., 2019)
*Heteropyxis natalensis* Harv.	Laventelboom, Lavender tree, Inkunzi	NT
*Leonotis leonurus* (L.) R. Br.	Wilde dagga, Wild dagga, Umhlahlampetu, Lebake, Umunyane	NT
*Lippia javanica* (Burm.f.) Spreng	Koorsbossie, Fever tea, Mumara, Musukudu, Inzinziniba, Umsuzwane	NT
*Mentha longifolia* (L.) L.	Kruisement, Wild mint, Koena-ya-thaba, Inixina, Ufuthanen lomhlange	NT
*Myrothamnus flabellifolia* Welw.	Bergboegoe, Resurrection plant, Uvukwabafile	NT
*Myrsine melanophloeos* (L) R. Br.	Kaapse boekenhout, Cape beech, Isiqwane-sehlati, Umaphipha	NT
*Osmitopsis asteriscoides* Less.	Bels, Belskruie	NT
*Pelargonium sidoides* DC.	Rabas, Khoaara e nyenyane, Ikhubalo	EPs^®^ 7630 (commercial product prepared from *P. sidoides*): EC_50_ = 44.50 ± 15.84 μg/mL (HCoV 229E); CC_50_ >100 μg/mL (Caco-2 cells); SI > 2.3 (Michaelis et al., 2011)
*Pellaea calomelanos* (Sw.) Link	Hard fern, Lehorometso, Inkomankomo	NT
*Protea repens* L.	Suikerbos, Sugarbush	NT
*Prunus africana* (Hook.f) Kalkman	Rooistinkhout, Red stinkwood, Umkakase, Inyazongoma-elimnyana	β-sitosterol: EC_50_ = 1210 μM (HCoV-NL63) (Lin et al., 2005)
*Rauvolfia caffra* Sond.	Kinaboom, Quinine tree, Umhlambamase, Umhlambamanzi	Reserpine: EC_50_ = 3.4 μM (SARS-CoV); CC_50_ = 2.5 μM (Vero-cells); SI: 7.3 (Wu et al., 2004)
*Salix mucronata* (Thunb.)	Wilde wilger, Wild wilow	NT
*Scadoxus puniceus* (L.) Friis & Nordal	Rooikwas, Red paintbrush, Umphompo	NT
*Searsia undulata* (Jacq.) T. S. Yi, A.J.Mill. & J. Wen	Koeniebos, Kuni-bush, T’kuni	NT
*Securidaca longipedunculata* Fresen.	Krinkhout, Violet tree, Mpesu	NT
*Siphonochilus aethiopicus* (Schweinf.) B.L.Burtt.	African ginger, Isiphephetho, Indungulo	NT
*Tarchonanthus camphoratus* L.	Wildekanferbos, Wild camphor bush, Sefehla, Umgebe, Mofahlana, Mohata, Mathola	NT
*Tetradenia riparia* (Hochst.) Codd.	Watersalie, Ginger bush, Iboza	NT
*Thesium hystrix* A.W. Hill	Kleinswartstorm	NT
*Tulbaghia violacea* Harv.	Wilde knoffel, Wild garlic, Isihaqa	NT
*Viscum capense* L. f.	Lidjiestee, Cape mistletoe	NT
*Withania somnifera* (L.) Dunal	Geneesblaarbossie, Winter cherry, Bofepha, Ubuvuma, Ubuvimbha	NT
*Xerophyta retinervis* Baker	Bobbejaanstert, Monkey’s tail, Isiphemba, Isiqumama	NT
*Zanthoxylum capense* (Thunb.) Harv.	Kleinperdepram, Small knobwood, Monokwane, Umlungumabele, Umnungamabele	NT
*Zingiber officinale* Roscoe	Gemmer, Ginger	NT
*Ziziphus mucronata* Willd.	Blinkblaar-wag-’n-bietjie, Buffalo thorn, Mokgalo, Umphafa, Umlahlankosi	*Z. jujuba* cyclopeptide alkaloids Jubanine H: EC_50_ = 4.49 ± 0.67 μM (PEDV, CoV); CC_50_ = 211.26 ± 29.64 μM (Vero cells); SI = 47.11 ± 0.49 Nummularine B: EC_50_ = 6.17 ± 0.50 μM (PEDV, CoV); CC_50_ = 165.30 ± 16.49 μM (Vero cells); SI = 26.75 ± 0.54 (Kang et al., 2015)

The plants have been selected from Medicinal Plants of South Africa (Van Wyk et al., 2009) and other resources, ^#^Not tested (selected based on traditional usage), ^+^50% effective concentration, ^++^50% cytotoxic concentration; ^##^Selective index (CC_50_/EC_50_), ^+++^50% inhibitory concentration.

*Artemisia afra*, has not been tested for its inhibitory potential against coronaviruses, however, a closely related species, *A. annua*, was able to inhibit SARS-CoV BJ-001 viral replication in Vero cells, with an EC_50_ value of 34.5 ± 2.6 μg/mL. Although these are two different species, it has been shown that within the *Artemisia* genus, many compounds are conserved; however, it is the small chemical nuances and profile that have a large effect on the biological activity (Abad et al., 2012). Medicinal plants species that are closely related may also produce similar or chemically similar compounds responsible for their biological activity (Nigam et al., 2019). This forms the basic definition for chemotaxonomy, which is the “closely related plants contain the same or similar chemical profiles” (Hao and Xiao, 2020). As an example, in a review article published by da Silva Mendes et al. (2020), many aspects of the *Cissampelos* genus were investigated, including the ethnobotanical aspects, isolated phytochemicals, and biological activity of the different species. Most of the biological activity described to species within the *Cissampelos* genus is attributed to the presence of alkaloids. The review, furthermore, describes the presence of similar compounds within different *Cissampelos* species. Many biological activities are attributed to warifteine, including results from clinical studies, and this compound was isolated from both *Cissampelos ovalifolia* and *Cissampelos sympodialis* (da Silva Mendes et al., 2020). Another example is the Southern African species, *Ziziphus mucronata*, which has not been investigated for its antiviral activity; however, cyclopeptide alkaloids isolated from *Z. jujuba* showed inhibition of a porcine-related coronavirus (porcine epidemic diarrhea virus (PEDV)), with SI values ranging from 7.98 to 47.11 on Vero cells (Kang et al., 2015).

Helichrysetin, a compound found within numerous *Helichrysum* species was able to inhibit MERS‐CoV 3CL^pro^ (Jo et al., 2019). A commercial product from *Pelargonium sidoides*, EPs^®^ 7630, showed a low selectivity index of 2.3 when tested against the human coronavirus strain 229E in Caco-2 cells. Two significant compounds identified within the traditionally used plants have been investigated for their potential against coronaviruses. *β*-Sitosterol, present in *Dodonaea viscosa* and *Prunus africana*, showed an EC_50_ value of 1210 μM against human coronavirus (HCoV-NL63) (Lin et al., 2005). Reserpine, a major constituent of *Rauvolfia caffra*, inhibited SARS-CoV viral replication with an EC_50_ value of 3.4 μM, CC_50_ value of 2.5 μM, and SI value of 7.3 (Wu et al., 2004). The further testing of the listed plant species could potentially identify a lead candidate for the treatment of COVID-19.

## Discussion

The COVID-19 pandemic has resulted in numerous clinical trials to evaluate whether existing drugs can be repurposed for the potential treatment of COVID-19. Studies have led to the following conclusions; treatment of COVID-19 might not be efficient if an antiviral drug alone is used, although small scale studies have shown some promise, larger-scale *in vivo* clinical studies are required to effectively evaluate the efficacy and safety of drugs. Furthermore, it is crucial to include placebo controls to adequately evaluate the potential benefit of a drug. Despite the publicity surrounding the drug, hydroxychloroquine as a potential treatment for COVID-19, RECOVERY has recently concluded that it has no beneficial effect toward severe cases of COVID-19 patients and therefore have stopped recruiting patients for clinical trials using hydroxychloroquine. However, treatment of patients with a combination of interferon beta-1b, lopinavir-ritonavir, and ribavirin was shown to be effective in alleviating COVID-19 symptoms and shortening virus shedding; however, this study lacked the required placebo control, and therefore, no conclusion can be made with regard to the therapeutic effect against COVID-19. Additional studies are required to substantiate these findings. *In vitro* studies showed that ivermectin was able to significantly reduce viral replication; however, no clinical trials have been completed to substantiate these results.

Studies have recently started to focus on the infection of SARS-CoV-2 in several other organs such as the heart, kidney, and liver, which widely express the ACE2 receptor, thereby leading to multiple organ toxicity and not only the severe infection of the lungs. Multiple studies have reported an increased inflammatory response in the endothelium, apoptotic bodies present within the heart, lungs, and small bowel; endotheliitis in the lungs heart, kidney, and liver; and necrosis of liver cells, which further suggests that antiviral treatment alone will not be sufficient and that a combination of drugs, including anti-inflammatories might be more effective, as shown in the preliminary study by Hung et al. (2020) (Hung et al., 2020). Additionally, the use of hepatoprotective drugs might be beneficial as Xu et al. (2020) reported that a COVID-19 patients’ liver tissue showed moderate microvesicular steatosis, which may have been due to COVID-19 infection or was due to drug-induced damage (Xu et al., 2020).

There have been countless studies where plant extracts and isolated compounds have been tested for activity against several strains of human coronaviruses. In this review, it was noted that extracts and compounds have been tested mainly against various target proteins of the coronaviruses such as protease activity (3CL^pro^), RNA-dependent RNA polymerase (RdRp), and papain-like proteinase (PL^pro^). These target proteins are critical for viral replication and infection in the host cell, thereby providing valuable targets for potentially inhibiting these processes. Cell culture–based techniques for testing the potential antiviral activity have been developed, which focus on screening of samples as potential viral inhibitors in an intracellular assay rather than testing activity using biochemical assays involving specific viral enzymes as mentioned above. There are several techniques that can be used to determine the antiviral activity of a sample. As an initial assay, the cytopathic effect (CPE) assay is most often used, which determines the ability of samples to prevent the virus from causing a cytopathic effect in the host cell. This also involves determining the potential toxicity of the sample against the host cell line used to perform the assay, which is most often depicted as the concentration required to cause toxicity to 50% of the host cells (CC_50_). The CPE assay is frequently followed by the viral reduction assay, which determines whether a sample is able to inhibit viral production in the host cell, post-infection. Additionally, the virucidal assay is used to determine the ability of a sample to kill the virus extracellularly before it infects the mammalian host cell line. This can be performed in a time-dependent manner in order to establish the shortest time necessary for the sample to display inhibition of viral infectivity. This can also include determining whether a sample is able to inhibit viral attachment and inhibit viral entry into the host cells (Lalani et al., 2020). The plaque assay was adopted for reliable determination of the titers of a wide variety of viruses. Each infectious particle produces a circular zone of infected cells, known as a plaque, which can be visually observed. This assay can only be performed using viruses that cause visible damage to the host cell (Flint et al., 2009). In a recent publication by Harcourt et al. (2020), it was shown that SARS-CoV-2 was not compatible with human lung adenocarcinoma (A549) cells and was able to moderately replicate in human liver (HUH7.0) and human embryonic kidney (HEK-293T) cells and was not able to replicate in big brown bat kidney (EFK3B) cells. However, results suggested that the best candidate for viral amplification and quantification was the VeroE6 cell line, which is widely used as a host cell line for antiviral studies (Harcourt et al., 2020).

When identifying a potential lead candidate with antiviral activity, pre-clinical toxicity studies are important to establish the margin of safety and to efficiently consider the risk-benefit of a proposed drug. The antiviral activity of a sample is determined by the 50% effective concentration (EC_50_), which is the concentration required to inhibit 50% viral replication/production using cellular-based assays or by the IC_50_ in assays where viral enzymes are targeted, such as proteases and polymerases. The overall therapeutic activity can be determined by calculating the selectivity index, which is defined as the ratio of the CC_50_ to the 50% concentration needed to inhibit viral replication (EC_50_). This provides valuable information on whether a sample is inhibiting viral replication without killing the host cell. Therefore, SI values that are >1 indicate that the inhibition is targeted toward viral replication and are less cytotoxic toward the host cell; therefore, the higher the SI value, the better the sample. There are no guidelines or cut-off values for an acceptable or appropriate SI value. It has been recommended that an SI value greater than 10 should be considered a good candidate. However, other factors, including the pharmacokinetic profile and drug delivery systems, can be used to mitigate associated toxicities. Extracts with a selectivity index of <10 should either undergo fractionation or purification to identify if a bioactive compound has increased therapeutic activity. When referring to *in vivo* animal studies it is denoted as the therapeutic index, where 50% lethal dose (LD_50_), which is determined from toxicity studies in animal models, is used instead of CC_50_ values obtained from *in vitro* toxicity studies. The therapeutic efficacy, in other words, described the margin of safety of a sample, compound, or drug (Abughazaleh and Tracy, 2014). However, there is no set guideline on defining whether a calculated selectivity index depicts significant therapeutic activity or not. Feng (2018) discussed that safety margins differ depending on the severity of a viral disease, where drugs used to treat acute diseases, such as Ebola, will differ in safety criteria compared to chronic viral infections, such as HIV (Feng, 2018). Muller and Milton (2012) further describe that when assessing the therapeutic efficacy of a drug, the risk-benefit analyses should be used, taking into account toxic effects that appeared frequently in clinical trials and not placing too much emphasis on rare toxic effects, which were only reported in large scale studies (Muller and Milton, 2012).

As mentioned, there are various reports of activity on the proteases and other molecular targets, although many lack the proper hypothesis, experimental design, and justification for the conclusion. The criteria for the effective identification of enzymatic inhibitors should be three-fold, namely, specificity, concentration-response, and kinetic characteristics. Firstly, a study needs to provide sufficient evidence to indicate that the enzyme activity is specific to the selected target. This can be achieved by testing on non-related targets and by screening the compounds for Pan-assay interference (PAINS) to rule out false positives. Secondly, the concentration range and response need to be appropriate, relevant, and realistic for the test system. A single test concentration or exorbitantly high concentration is not sufficient and appropriate to confirm enzymatic inhibition. Lastly, an attempt should be made to identify the kinetic properties and mode of inhibition (competitive, non-competitive or un-competitive) through appropriate kinetic assessment.

The most prominent compounds identified in this review, are the abietane diterpenoids, triterpene glycosides and chalcones. Since these compounds possess medium polarity, these can be easily extracted with organic extracts such as dichloromethane, chloroform, ethyl acetate and alcohols. These are common classes of natural products occurring abundantly in several plant species including South African plants. However, clinical toxicity and efficacy trials are still necessary for each of the identified natural products. In this review, we also attempted to identify potential leads from a Southern African perspective. Two propositions were evident. Firstly, these plants are highly under-investigated. Secondly, the “related-species” approach can be useful in selecting the initial candidates for further testing. This approach might be somewhat speculative but should not be overlooked. Related species may well have similar chemical profiles or slightly varying constituents that can have a beneficial effect on the biological activity. Lastly, bioprospecting, access, and benefit-sharing related to traditional knowledge on the usage of medicinal plants for COVID-19 pathogenesis and/or related symptoms should beincluded in the study design. Should any plant samples or related natural products show potential for commercialization (pharmaceutical or nutritional supplement development), a bioprospecting permit should be obtained in the respective countries. Although COVID-19 is considered a novel viral disease many plant species mentioned in this review article, have a direct link to the traditional usage of the plants for COVID-19 related symptoms. The Nagoya protocol guidelines, as well as national and international regulations, should be followed for commercialization purposes to ensure the knowledge holders and communities benefit.

## Conclusion

The current COVID-19 pandemic, caused by SARS-CoV-2, is a major global health concern and there is a social and ethical responsibility for communities and scientists around the world to work together to effectively combat the disease. In this review, we investigated the current state of natural products research to identify potential anti-coronaviral compounds, current drugs being used and potential lead candidates for the treatment of COVID-19, specifically from plants. Lycorine, savinin, and 8-hydroxe were the most prominent compounds identified that showed high selectivity. Southern Africa boasts a large biodiversity and subsequent natural products diversity, providing a substantial source of candidates to be screened against SARS-CoV-2 and its protein targets. Combining this with an ethnobotanical approach, it is evident that there exists a vast potential to discover new antiviral compounds. Several techniques have been used to identify potential lead from natural sources; these include the ethnopharmacological approach, similarities in previously identified active compounds, and computational models such as molecular docking. However, selecting compounds for further clinical assessment should be carefully considered, and the necessary *in vitro* and *in silico* experimental evidence needs to be conclusive. Finally, matters relating to bioprospecting and the fair and equitable sharing of benefits should be included in projects that are related to traditional knowledge systems.

## Author Contributions

All listed authors contributed equally to this review.

## Conflict of Interest

The authors declare that the research was conducted in the absence of any commercial or financial relationships that could be construed as a potential conflict of interest.

## Abbreviations

3CL^pro^: Coronavirus main protease 3CL^pro^; ACE2: Angiotensin-converting enzyme 2; COVID-19: Coronavirus disease of 2019; EC_50_: Fifty percent effective concentration; FDA: Food and drug administration; HCoV: Human coronavirus; IC_50_: Fifty percent inhibitory concentration; ICMR: Indian Council of Medical Research; IFN: Interferons; IFNAR: Interferon alpha-receptor; MERS-CoV: Middle East respiratory syndrome-related coronavirus; NF-κβ: nuclear transcription factor; Nsp1: Nonstructural protein 1; NSP13: Non-structural protein 13; ORF7a: Open reading frame 7a; PAINS: Pan assay interference compounds; PL^pro^: Papain-like protease; RBD: Receptor binding domain; RdRp: RNA-dependent RNA polymerase; RNA: Ribonucleic acid; S protein: Viral spike glycoprotein; SARS-CoV: Severe acute respiratory syndrome-related coronavirus; SARS-CoV-2: Severe acute respiratory syndrome-related coronavirus-2; SI: Selective index; TLR: Toll-like receptors; TMPRSS2: Transmembrane protease, serine 2; TNF-α: Tumor necrosis factor-alpha; WHO: World Health Organization.
